# Carotid ultrasound phenotypes in vulnerable populations

**DOI:** 10.1186/1476-7120-4-44

**Published:** 2006-11-13

**Authors:** Silvia A Riccio, Andrew A House, J David Spence, Aaron Fenster, Grace Parraga

**Affiliations:** 1 Imaging Research Laboratories, Robarts Research Institute, 100 Perth Drive, London, Ontario, N6A 5K8, Canada; 2 Department of Medical Biophysics, The University of Western Ontario, London, Ontario, N6A 5C1, Canada; 3 Department of Medicine, The University of Western Ontario, Department of Nephrology, University Hospital, London Health Sciences Centre, 339 Windermere Road, London, Ontario, N6A 5A5, Canada; 4 Stroke Prevention and Atherosclerosis Research Centre, Robarts Research Institute, 1400 Western Road, London, Ontario, N6A 5K8, Canada; 5 Graduate Program in Biomedical Engineering, The University of Western Ontario, London, Ontario, N6A 5B9, Canada

## Abstract

**Background:**

Biomarkers of carotid atherosclerosis range from those that are widely available and relatively simple to measure such as serum cholesterol levels, and B-mode Ultrasound measurement of intima media thickness (IMT) to those that are more complex and technologically demanding but perhaps potentially more sensitive and specific to disease such as total plaque volume and total plaque area measured from 3-dimensional ultrasound images. In this study we measured and compared intima media thickness (IMT), total plaque volume (TPV) and total plaque area (TPA) in two separate populations, both vulnerable to carotid atherosclerosis.

**Methods:**

In total, 88 subjects (mean age 72.8) with carotid stenosis of at least 60%, based on a peak Doppler flow, and 82 subjects (mean age 60.9) with diabetic nephropathy were assessed in a cross-sectional study. Conventional atherosclerotic risk factors were examined and the associations and correlations between these and carotid ultrasound phenotypes measured from B-mode and 3-dimensional ultrasound images were assessed.

**Results:**

IMT and TPV were only modestly correlated in the two separate populations (r = .6, p < .01). ANOVA analyses indicated that both IMT and TPV were significantly associated with age (p < .001) and Framingham score (p < .05), but only TPV was associated with diabetes (p < .001) and presence of plaque ulcerations (p < .01)

**Conclusion:**

IMT and TPV were modestly correlated in a diabetic patient population and only TPV was associated with diabetes and the presence of plaque ulcerations in a diabetic population and carotid stenosis group. The 3-dimensional information provided by TPV can be critically important in unmasking association with risk factors not observed with less complex single-dimension assessments of carotid atherosclerosis such as those provided by IMT.

## Background

Measurements of atherosclerosis range from clinical events such as myocardial infarction and stroke, and biomarkers such as blood pressure, serum cholesterol and triglycerides levels, to measurements acquired with non-invasive imaging modalities such as computed tomography (CT), magnetic resonance imaging (MRI) and ultrasound (US). Non-invasive imaging measurements can be considered as physical characteristics or phenotypes that can be measured in specific subjects and populations. Such physical characteristics or phenotypes that are shown to correlate with disease activity can be considered to be disease biomarkers, and those biomarkers that can be shown to correlate with clinical outcomes are considered as surrogate endpoints. Non-invasive imaging phenotypes are also of particular interest as they may reflect different stages of or different underlying mechanisms that contribute to atherogenesis; some of these phenotypes directly reflect changes in plaque and atherosclerotic vessel architecture providing a distinct advantage over biochemical biomarkers from blood samples. Quantitative assessment of different non-invasive imaging phenotypes in different vulnerable patient populations is an important approach to understanding natural disease progression and treatment-related disease regression.

An emerging imaging phenotype of atherosclerosis is carotid total plaque volume (TPV), measured with three-dimensional ultrasound and specialized image processing software [1]. The current widely-accepted non-invasive measurement of atherosclerosis based on measurement of one-dimensional thickening of the artery wall is intima-media thickness (IMT). While IMT measurements have been validated in many studies, it is important to acknowledge that many distinct cellular, biological and pathological pathways and mechanisms may be reflected by the measurement. For example, a measurement of IMT may represent hypertensive medial hypertrophy [2,3], compensatory intimal thickening due to mechanical forces of blood flow [4,5], or the initial "fatty streak" stage of atherosclerosis that involves accumulation of macrophage foam cells in the artery wall [6]. Moreover, the intima and media also change over time in response to a variety of factors, some of these not necessarily related to atherosclerotic plaque formation and progression. Carotid TPV is a direct 3-dimensional (3D) measurement of atherosclerotic plaque burden, and directly assesses atherogenesis when lesions develop beyond the initial fatty streak to the atheroma classification. The direct measurement of plaque volume itself would be expected to be highly sensitive and specific to atherosclerosis disease progression. Indeed, three-dimensional volumetric measurements of plaque provide enhanced "dynamic range" over the one-dimensional IMT measurement [7].

In this study we aimed to measure and compare total plaque volume (TPV), total plaque area (TPA) and intima-media thickness (IMT) in the carotid arteries, as well as previously established biomarkers, in two special and vulnerable populations: middle-aged patients with diabetic nephropathy and elderly patients with asymptomatic carotid stenosis of at least 60%. Imaging measurements of atherosclerosis that we continue to develop and validate are assessed in these populations to better understand the associations of these with one another and with risk factors related to atherosclerosis progression.

## Methods

### Study sample

One study population consisted of 88 subjects with internal carotid artery stenosis (carotid stenosis population) of at least 60%, based on a peak Doppler flow velocity cut-off established by the North American Carotid Endarterectomy Trial (NASCET) [8]. These subjects regularly attended the Stroke Prevention and Atherosclerosis Research Centre (SPARC) at Robarts Research Institute; they were participants in a long-term study to investigate imaging methods that may reveal vascular phenotypes associated with cardiovascular events. Although some subjects had a history of transient ischemic attacks, all patients had been asymptomatic for at least 18 months prior to beginning the study. The comparison population consisted of 82 patients with clinical diagnosis of diabetic nephropathy (diabetic population). Subjects were recruited among patients who regularly attended the Nephrology or Diabetes Clinics of the London Health Sciences Centre and participated in a long-term placebo-controlled trial investigating the effects of vitamin therapy on plasma homocysteine reduction. Both cohorts underwent 3D and standard B-mode ultrasound scans of the carotid arteries. All subjects provided written informed consent to protocols approved by The University of Western Ontario Health Sciences Research Ethics Board.

### Risk factors and biochemical determinations

Clinical data collected and used in the analysis of both populations were age, sex, body mass index (BMI), plasma cholesterol, low-density lipoproteins (LDL), high-density lipoproteins (HDL) and triglycerides, blood pressure (BP), number of plaque ulcerations, diabetes status and smoking history. Age, sex, height and weight were self-reported. For both cohorts, blood pressure was measured once in one arm (the one arm previously determined to have the highest pressure), as many as three times. The average of multiple measurements was used. Subjects with physician-diagnosed diabetes were considered to be diabetic. Smokers were defined as those who currently smoked or those who had stopped smoking less than one year prior to the ultrasound scan. Blood for biochemical determinations was collected after a 12-hour fast. Plasma measurements included triglycerides, total cholesterol, HDL and LDL. Framingham risk scores [9], defined as the percent risk of experiencing a coronary heart disease event within 10 years, were calculated for each patient. Coronary heart disease events were defined as myocardial infarction, angina or sudden death. The risk score calculation included the following parameters: age, sex, LDL, HDL, BP, presence of diabetes and smoking status. In the diabetic population, for 10 subjects for whom LDL data was not available, an alternative calculation with total cholesterol values was used. A Framingham risk score was not calculated for four subjects who did not have HDL results. Clinical data for the asymptomatic stenosis cohort were collected up to six months prior to ultrasound scanning, whereas clinical data for the diabetic population were collected at the time of the ultrasound scans.

### General ultrasound logistics

Ultrasound (US) images were acquired with an HDI 5000 duplex machine and an L12-5 transducer (both from Advanced Technology Laboratories, Bothell, WA USA). A single experienced sonographer acquired images suitable for determination of IMT, TPA and TPV.

### Intima-Media Thickness (IMT) data acquisition and measurement

Ultrasound images were acquired from an anterolateral longitudinal view and saved to S-VHS tapes. The sonographer used the minimum gain necessary to clearly visualize lumen-intima and media-adventitia echoes, which are of best quality when the image plane is parallel to the carotid artery axis. These views were reviewed using an image processing board and a specialized recorder with digital memory permitting digitization of a full video frame in "still" mode. The resultant digitized ultrasound images, captured at end-diastole, were analyzed using Prowin [10] edge-detection software. Two to six points were placed manually on each of the lumen-intima and media-adventitia boundaries on the far wall of the artery using a mouse driven cross-haired cursor. The software algorithm functions by deleting weak edge points and filling boundary gaps by linear interpolation. Mean IMT is then computed from 80 to 120 measurements over a 10-mm span ending 5 mm proximal to the transition between the common carotid and bulb regions. Mean IMT was the average of the measurements from the left and right carotid arteries. Measurements were made by a single reader, (SAR), and resulted in an intra-reader intraclass correlation (ICC) of 0.92. The ICC was determined from 20 images measured twice, two weeks apart.

### Total Plaque Area (TPA) measurement

Plaque area measurements were made by a trained sonographer on magnified, longitudinal views of the common carotid arteries in real time. The patient was scanned until the largest extent of a plaque could be seen, and the image was used to outline plaque with the use of a trackball and the area was immediately calculated by the microprocessor in the scanner. Plaque was defined as a local thickening of the intima layer of greater than 1 mm [11]. This was repeated for all plaques in the common, external and internal carotids. TPA was the sum of all plaques between the clavicle and the angle of the jaw in both left and right carotid arteries. Measurements were made by one reader, and resulted in an ICC of 0.94. This ICC was determined by scanning the same 25 subjects twice, one week apart.

### Total Plaque Volume (TPV) measurement

3D US images were viewed and analyzed using 3D Quantify software [12]. The protocol for measuring plaque volume involved outlining the vessel on sequential transverse slices and using the contours to locate the carotid bifurcation, which was then marked with a single point using a mouse driven cursor. A 2.0 cm axis was then set along the length of the vessel, +/- 1.0 cm from the carotid bifurcation. Plaque was outlined on sequential transverse slices by manually clicking a mouse driven cursor at regular intervals around the plaque. Measurements were made at 1.0 mm increments along the axis of the vessel, an interslice distance which has been shown to optimize accuracy and reliability [1]. TPV was determined by summing the calculated areas in each slice over the 2.0 cm region and multiplying by the interslice distance. All measurements were made by a single reader. Intra-reader ICC was 0.91, based on five images read five times at one-week intervals.

### Plaque ulceration scoring

Plaque ulcerations in the common and internal carotid arteries were scored by a single observer. Plaque ulcerations were identified as focal defects in plaque surface of at least 0.5 mm in depth, length and width that were visible from multiple angles. Subjects were scored as positive if at least a single ulceration was observed in the 3D US image.

### Statistical analyses

Data analysis was conducted with statistical software SPSS version 12.0 (SPSS Inc., 2003). Associations between the different ultrasound measures of atherosclerosis were determined using the Pearson product-moment correlation. For the asymptomatic stenosis population, the correlation was determined between TPA and TPV. For the diabetic nephropathy population, correlations were determined between IMT, TPA and TPV. Distributions were significantly non-normally distributed and therefore, IMT TPA and TPV variables were transformed and subjected to analysis of normality. After transformation, the inverse of IMT, and cubed root of both TPV and TPA were normally distributed. The transformed variables were used for the analyses. Simple linear regressions were performed predicting the inverse of IMT, the TPV and TPA transformed by cube root, using a number of dichotomous and continuous predictors: age, sex, BMI, BP, number of ulcerations, diabetes and smoking history, and Framingham score. This analysis was conducted for the populations as a whole, as well as for subpopulations characterized by sex and, in the diabetic cohort, diabetes type. A significant result indicates that there is a significant difference between the categories from the dichotomous and continuous variables with respect to the phenotypes measured.

## Results

### Baseline demographic features

Clinical attributes for the two subject populations, as a whole and by sex, are provided in Table 1. The two populations differed significantly in both discrete and quantitative traits. The mean age of the asymptomatic stenosis population was 72.8 ± 8.6 years, which was significantly older than the mean age of the diabetic nephropathy population (60.9 ± 11.2 years). TPA and TPV for the stenosis population were both significantly greater than the corresponding measurements in the diabetic population. Mean TPA was 2.5 times greater and mean TPV was 4.2 times greater in the carotid stenosis cohort as compared to the diabetic cohort. Mean IMT, which was not measured in the carotid stenosis population, was 0.87 ± 0.17 mm in the diabetic population. The populations also differed with respect to plaque ulcerations, in that just over half of the carotid stenosis population showed 3D US evidence of at least a single plaque ulceration. The proportion of diabetics in each population was significantly different with 17.0% of the stenosis population reporting physician-diagnosed type 2 diabetes and the diabetic nephropathy population consisted of a combination of type 1 and type 2 diabetics, at 17.1% and 82.9%, respectively. The diabetic population had significantly BMI, total cholesterol and triglycerides than the carotid stenosis population. Although the diabetic nephropathy cohort was much younger, Framingham scores were greater than two-fold higher as compared to the carotid stenosis population. Significant differences between males and females in each population were identified for smoking. Also, in the carotid stenosis population, females had both lower TPV and TPA than males.

**Table 1:** Baseline demographics of 88 asymptomatic carotid stenosis and 81 diabetic nephropathy study subjects.

	**Asymptomatic Stenosis All**	**Male**	**Female**	**Diabetic Nephropathy All**	**Male**	**Female**
***Discrete Traits***						
No.	88	63	24	82	55	27
Female	27.3%			32.9%		
Current Smoking	19.3%	23.4%t	8.3%t	13.4%	16.4%	7.4%
Type 1 Diabetes**	0.0	0	0	17.1%	14.5%	22.2%
Type 2 Diabetes**	17.0%	18.8%	12.5%	82.9%	85.5%	77.8%
Cholesterol Lowering Treatment n(%)	80.5 (82)	83.1	73.9	70.7		
Hypertension Treatment n (%)	92.7 (82)	91.5	95.7	98.8		
≥ 1 Ulceration (%)**	51.1	51.6	50.0	11.0	10.0	11.1
**Quantitative Traits (mean+/- SD**						
Age (y)**	72.8 ± 8.6	73.1+/-8.2	72.0+/-9.6	60.9 ± 11.2	61.2+/-11.2	60.3+/-11.4
BMI (kg/m^2^)**	26.4 ± 4.0 (81)	26.6 +/-3.6	26.0+/-5.0	33.2 ± 5.9	32.6+/-5.6	34.3+/- 6.6
Total Chol (mmol/L)**	4.38 ± 0.94	4.27+/-0.96	4.64+/-0.85	4.95 ± 1.56 (80)	4.65+/-1.34t	5.57+/-1.81t
Triglycerides (mM)**	1.46 ± 0.57	1.40+/-0.56	1.64+/-0.56	2.64 ± 1.81 (78)	2.43+/-1.36	3.06+/-2.47
Framingham Score (%)**	8.3 ± 4.4	8.0 +/-4.1	9.3+/-5.2	19.6 ± 11.4 (78)	21.3+-12.6t	16.2+7.9t
Carotid IMT (mm)	ND	ND	ND	0.87 ± 0.17	0.88+/-0.15	0.84+/-0.20
Carotid TPA (cm^2^)**	3.4 ± 1.8	3.8+/-1.8**	2.4+/-1.5**	1.3 ± 1.2	1.4+/-1.0	1.3+/-1.5
Carotid TPV (mm^3^)**	690 ± 330	790+/-330**	420+/-150**	160 ± 170	180+/-170	120+/-150

** difference is significant at the 0.01 level; t difference is significant at 0.05 level IMT, mean carotid intima-media thickness of measured right and left carotid IMT; TPV, total volume of carotid plaque burden: sum of plaque volume from right and left carotid; TPA, total plaque area ; Type 1 and Type 2 diabetes refers to physician-diagnosed diabetes; Lipid-lowering and antihypertensive medication history was retrieved from physician chart; The numbers in brackets indicate the sample size for which the given trait was calculated due to unavailable data.

### Carotid ultrasound data

**Figure 1:**
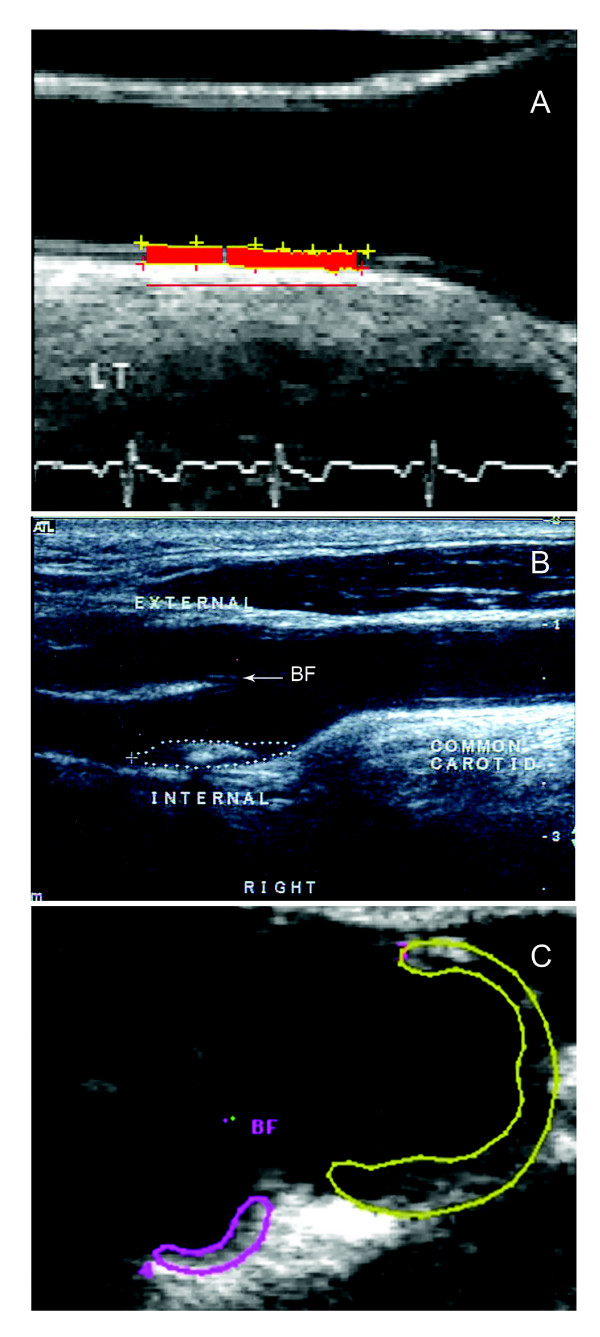
**Carotid atherosclerosis imaging phenotypes**. A) IMT measurement from a B-mode ultrasound image in the longitudinal view with the carotid bifurcation identified as BF. B) TPA measurement from a longitudinal B-mode US image with the outlined plaque area shown in white. C) cross-sectional or axial view of the internal and external carotid artery with manual TPV measurements in both branches of the artery and the bifurcation marked BF.

Figure 1 shows representative ultrasound images for subjects evaluated in this study. Figure 1A shows an IMT measurement from a B-mode ultrasound image in the longitudinal view with the carotid bifurcation clearly identified (BF). As shown, IMT measurements are made in the longitudinal view on the far wall (from the ultrasound transducer) and the semi-automated 1-dimensional measurement is defined by the red line between the intima-lumen boundary and the media-adventitia boundary. Figure 1B shows a TPA measurement from a B-mode US image with the manually outlined plaque area shown in the longitudinally view. Figure 1C shows a cross-sectional or axial view of the internal and external carotid artery with manual TPV measurements in both branches of the artery and the bifurcation identified as BF.

IMT data are provided in Table 1 for the diabetic nephropathy population. There were no differences in IMT measurements for males and females. TPA and TPV measurements for both the carotid stenosis and diabetic nephropathy population are also provided in Table 1 and for the carotid stenosis population only, TPA and TPV measurements were greater in males than in females.

**Figure 2:**
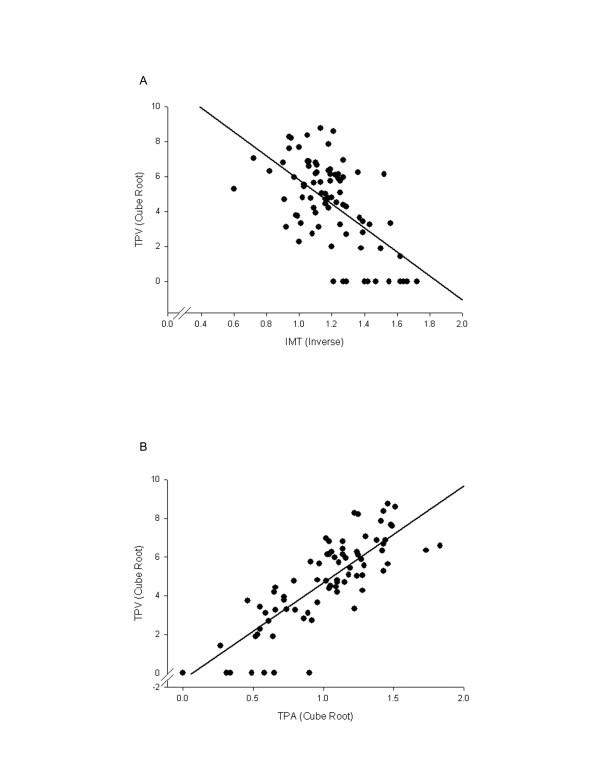
**Ultrasound phenotype correlations for diabetic nephropathy population**. A) Pearson Correlation for IMT and TPV in diabetic subgroup and B) Pearson Correlation for TPA and TPV in diabetic subgroup.

Pearson correlation coefficients between transformed IMT, TPA and TPV are shown in Table 2. For the carotid stenosis population, correlations between TPA and TPV were modest and significant. For the diabetic nephropathy population (see Figure 2), IMT correlations with TPA and TPV were modest (r = -.600 and -.606 respectively) and significant while TPA-TPV correlations where high (r = .851) and significant.

**Table 2:** Carotid ultrasound phenotype correlations

**Carotid Stenosis**	**IMT**	**TPA**	**TPV**
**IMT**			
**TPA**		1	0.598
**TPV**			1

**Diabetic Nephropathy**	**1/IMT**	**TPA**^1/3^	**TPV**^1/3^

**IMT (inverse)**	1	-0.606	-0.600
**TPA (cube root)**		1	0.851
**TPV (cube root)**			1

All correlations significant at the 0.01 level. TPA and TPV in the carotid stenosis population were normally distributed. Inspection for skewness and kurtosis revealed significantly non-normal distributions for IMT, TPA and TPV in the diabetic nephropathy population. These variables were transformed to produce distributions that did not deviate significantly from normal. Inverse transformation was used for IMT (1/IMT) and cube root transformation was used for area and volume measurements (TPA^1/3 ^and TPV^1/3^).

### Correlations with demographic features

As shown in Table 3, simple linear regressions were performed predicting the inverse IMT as well as TPV and TPA transformed by cube root, using a number of dichotomous and continuous predictors. ANOVA analyses indicated that transformed IMT was significantly associated with age (p < .001), diastolic BP (p < .01) and Framingham score (p < .05) for the diabetic population and TPV and TPA were both significantly associated with age (p < .001), diabetes (p < .001), presence of plaque ulcerations (p < .01) and Framingham score (p < .01). The same analysis of the carotid stenosis population revealed that both TPA and TPV were significantly associated with sex (p < .001) and presence of ulcerations (p < .01). In the younger diabetic population, all carotid ultrasound phenotypes were significantly associated with age and Framingham score, whereas in the older carotid stenosis population, only sex (male) and presence of ulcerations were significant predictors of TPA and TPV. Since there was a correlation between TPA and TPV and diabetes, subpopulations of the diabetic nephropathy cohort were assessed with respect to diabetes type. While mean age for type 2 diabetics (n = 68) was 63.8 ± 9.0 years, type 1 diabetics (n = 14) were significantly younger with mean age 47.0 ± 10.8 years. TPA and TPV for type 2 diabetics (1.46 ± 1.23 cm^2 ^and 187 ± 170 mm^3^, respectively) were significantly greater than for type 1 diabetics (TPA 0.74 ± 0.83 cm^2 ^and TPV 47.2 ± 55.6 mm^3^). The two subgroups, however, did not significantly differ in mean IMT. As this and previous work shows that age is predictive of TPA and TPV in younger populations, TPA and TPV were reassessed for correlations with of diabetes type while controlling for the mean age difference between type 1 and type 2 diabetics. In this analysis we found that TPV was not significant (p < 0.088) and neither was TPA (p < 0.959). As the TPV result approached significance, a sample size calculation was performed to determine how many individuals would be required to achieve significant differences (power of 0.8 and an alpha of 0.05) and this indicated that 216 subjects across diabetes types would be required to detect group differences when controlling for age.

**Table 3:** Determinants of carotid ultrasound traits

**Diabetic Nephropathy**				**Carotid Stenosis**		
Source of Variation	Degrees Freedom	F	P > F	Degrees of Freedom	F	P > F

*Dependent Variable: mean inverse carotid intima media thickness*						

Age	1	20.95	<0.001			
Sex	1	2.49	NS (0.11)			
BMI	1	0.28	NS (0.59)			
Diabetes	1	2.66	NS (0.10)			
Current Smoking	1	0.10	NS (0.75)			
Systolic BP	1	1.46	NS (0.22)			
Diastolic BP	1	10.34	<0.01			
Presence Ulcers	1	3.15	NS (0.07)			
Framingham Score	1	4.55	<0.05			
						

*Dependent Variable: mean cubed root total plaque volume*						

						
Age	1	29.15	<0.001	1	0.67	NS (0.41)
Sex	1	3.44	NS (0.06)	1	29.94	<0.001
BMI	1	0.52	NS (0.47)	1	0.002	NS (0.96)
Diabetes	1	17.71	<0.001	1	0.09	NS (0.75)
Current smoking	1	0.01	NS (0.73)	1	0.25	NS (0.61)
Systolic BP	1	0.98	NS (0.32)	1	0.01	NS (0.91)
Diastolic BP	1	1.96	NS (0.16)	1	0.84	NS (0.36)
Presence Ulcers	1	7.55	<0.01	1	9.27	<0.01
Framingham Score	1	11.17	<0.01	1	0.12	NS (0.72)
						

*Dependent Variable: mean cubed root total plaque area*						

						
Age	1	22.20	<0.001	1	0.187	NS (0.66)
Sex	1	1.50	NS (0.22)	1	16.49	<0.001
BMI	1	0.59	NS (0.59)	1	0.11	NS (0.73)
Diabetes	1	6.16	<0.05	1	1.06	NS (0.30)
Current Smoking	1	0.04	NS (0.82)	1	2.65	NS (0.10)
Systolic BP	1	0.13	NS (0.71)	1	0.10	NS (0.74)
Diastolic BP	1	3.58	NS (0.06)	1	0.07	NS (0.78)
Presence Ulcers	1	15.01	<0.001	1	10.75	<0.01
Framingham Score	1	6.29	<0.05	1	0.10	NS (0.74)

## Discussion

IMT measurements has been used for many years in the assessment of carotid atherosclerosis; IMT image acquisition and analysis is relatively straightforward and provides a one-dimensional measure of carotid artery wall changes. However, different multi-dimensional methods of measuring atherosclerosis may potentially provide biologically distinct and perhaps more sensitive and specific measures of carotid atherosclerosis [13]. For example, in a previous study at our centre, we found that traditional risk factors accounted for 52% of variance of carotid plaque area [14] and 13% of variance of carotid stenosis, whereas O'Leary et al found that these accounted for 15–17% the variance of carotid IMT [15]. This suggests that the different phenotypes reflect different biological mechanisms related to atherosclerosis. IMT, for example, constitutes approximately 80% media and 20% intima [16]. Thus, IMT measurements represent medial hypertrophy in addition to atherosclerosis, introducing blood pressure-related effects as well as atherosclerosis-related effects in IMT measurement values

Hegele and collaborators first showed that different atherosclerosis phenotypes associate with different risk factors in a closed aboriginal population [17]. In their analysis of a subject population distinct from the two cohorts studied here, ultrasound phenotype correlations were similar to those found in our analysis of middle-aged diabetics and elderly with carotid stenosis; IMT was found to be associated with age and hypertension, and TPV with age and the presence of diabetes. Different associations between phenotypes with PPARG genotypes and PCK1 variants were also identified [17-20] and TPV in particular was increased over a seven year period for type 2 diabetic subpopulation of the cohort [7]. Our analysis of two additional vulnerable populations extends these landmark findings and further suggests that IMT and TPV may represent different stages of atherosclerosis and may also reflect different biological and genetic contributions to, or attributes of, the atherosclerotic process. The fact that in both populations, 2-dimensional and 3-dimensional ultrasound phenotypes associate significantly with plaque ulcerations also suggests these are related to plaque vulnerability in both middle-aged diabetics and elderly subjects with carotid stenosis.

Overall these results suggest that in different subject populations, both vulnerable to vascular disease, carotid ultrasound phenotypes associate differently with various risk factors. Differences were observed as well in that TPA and TPV in both populations were associated with ulcerations, whereas IMT was not associated with plaque ulcerations for the diabetic population where IMT was measured. Moreover, for the diabetic population, only TPA and TPV were significantly associated with diabetes type, and not IMT. This suggests that advanced plaque development that is reflected in measurements of TPA and TPV is associated with type 2 diabetes and that the presence of type 2 diabetes may play a role in mechanisms contributing to plaque burden, but not contributing specifically to IMT.

## Conclusion

Here we extend the first reported comparisons of imaging phenotypes of atherosclerosis by Hegele and collaborators [7,17-20] and report: 1) correlations between the ultrasound imaging phenotypes of atherosclerosis, 2) relationships between risk factors and US phenotypes, and 3) comparison of associations between US phenotypes in two significantly different vulnerable patient groups.

While the mean age of the carotid stenosis cohort was nearly 12 years older, mean TPA and TPV were 2.5 and 4.2 fold greater than in the younger diabetic cohort and over half the older cohort showed 3D US evidence of at least one ulcer. In the older stenosis population only, females had statistically significantly lower mean TPA and TPV compared to males. Although the diabetic nephropathy cohort was much younger, Framingham scores were greater than two-fold overall risk as compared to the older stenosis cohort.

Hegele and collaborators showed that for a middle-aged aboriginal population (Sandy Lake Canada) carotid IMT was associated with age and hypertension, whereas TPV was associated with age and the presence of diabetes. In our analysis, IMT was significantly associated with age, diastolic BP and Framingham score, but not diabetes. TPV and TPA were significantly associated with age and diabetes and with the presence of ulcerations and Framingham score. For the elderly population with significant carotid stenosis, both TPA and TPV were significantly associated with sex and presence of ulcerations. Such differences in risk factor and ultrasound phenotypes in different populations create a framework for understanding risk factor relationships in middle age and elderly populations

Because different phenotypes may be related to different biological and genetic factors, the use of these phenotypes as measurement tools may provide for a wide range of sensitivities and specificities to disease progression and regression that is population-dependent. While our sample sizes are small, our results suggest that while imaging measures themselves correlate similarly across diverse populations, a given imaging measures associates with different risk factors in different populations. We suggest that clinical studies that aim to use imaging measures as surrogate endpoints should measure specific biomarkers that are dependent on the treatment and specific population being studied. It may be necessary for specific new interventions to be targeted to specific study populations that are well characterized with respect to genotype, phenotype or other underlying characteristics. Study populations may need to be targeted based on the likelihood of well-characterized treatment effects that can be predicted in advance using the approaches we have taken here. While all of these issues require clarification in the future, it is clear that individual imaging measures such as IMT, TPA and TPV will require rigorous testing and validation in specific target populations before they can be used as surrogate endpoints in treatment studies.

## Competing interests

The author(s) declare that they have no competing interests.

## Authors' contributions

SAR measured all carotid US phenotypes and performed some of the statistical analyses. AAH trained the IMT observer, supervised the analysis of IMT, assisted in the final analysis of the data and helped draft the manuscript. AF participated in the analysis of the imaging data and assisted in the supervision of the imaging phenotype measurements. JDS participated in the design and analyses of the study. GP conceived the study, participated in its design and statistical analyses, analysed the statistical and some imaging data and drafted the manuscript. All authors read and approved the final manuscript.
